# Analysis of acute flaccid paralysis surveillance in Ethiopia, 2005-2015: progress and challenges

**DOI:** 10.11604/pamj.supp.2017.27.2.10694

**Published:** 2017-06-09

**Authors:** Ayesheshem Ademe Tegegne, Braka Fiona, Meseret Eshetu Shebeshi, Fasal Teshager Hailemariam, Aron Kassahun Aregay, Berhane Beyene, Eshetu Wassie Asemahgne, Daddi Jima Woyessa, Abyot Bekele Woyessa

**Affiliations:** 1 World Health Organization Country Office, Ethiopia; 2 World Health Organization Country Office, Nigeria; 3 WHO Inter-Country Support Team (IST), Harare; 4 Ethiopian Public Health Institute, Addis Ababa, Ethiopia

**Keywords:** Progress acute flaccid paralysis surveillance, case-based surveillance, non-polio AFP rate, two main indicators, non-polio enterovirus, performance indicators

## Abstract

**Introduction:**

Ethiopia joined the global effort to eradicate polio in 1996, and interrupted indigenous wild poliovirus transmission by December 2001. However, the country experienced numerous separate importations during 2003-2013. Sensitive Acute Flaccid (AFP) surveillance is critical to rule out undetected circulation of WPV and VDPVs.

**Methods:**

In this study described, we used a retrospective descriptive study design to characterize the surveillance performance from 2005 to 2015.

**Results:**

The none-polio AFP rate improved from 2.6/100,000 children <15 years old in 2005 to 3.1 in 2015, while stool adequacy has also improved from 78.5% in 2005 to 92 % in 2015. At the national level, most AFP surveillance performance indicators are achieved and maintained over the years, however, AFP surveillance performance at sub-national level varies greatly particularly in pastoralist regions. In addition, the minimum standard for non-polio enterovirus isolation rate (10%) was not achieved except in 2007 and 2009. Nevertheless, the proportion of cases investigated within 2 days of notification and the proportion of specimens arriving in good condition within 3 days to the laboratory were maintained throughout all the years reviewed.

**Conclusion:**

We found that the AFP surveillance system was efficient and progressively improved over the past 10 years in Ethiopia. However, the subnational AFP surveillance performance varies and were not maintained, particularly in pastoralist regions, and the non-polio enterovirus isolation rate declined since 2010. We recommend the institution of community-based surveillance in pastoralist regions and conduct detail review of the laboratory sensitivity and the reverse cold chain system.

## Introduction

Polio is a paralytic illness with a permanent disability, varying in severity from asymptomatic to severe, primarily affecting children below 5 years old. Only less than one percent of children infected with polio become paralyzed, and for every paralyzed child there are approximately 200 children infected and asymptomatic around [1]. The Global Polio Eradication Initiative(GPEI), the most astronomical immense public health program ever in the history of disease eradication, aimed to eradicate the disease by 2000 and has since revised the target for global certification by 2018 [2]. Sensitive Acute Flaccid Paralysis (AFP) surveillance is one of the critical strategies for polio eradication aiming at capturing a high number of AFP cases in children below 15 years old including those with polio. Poliomyelitis remains endemic before the endorsement of the polio eradication initiative in most countries in the African content, particularly in countries with difficult situations [3].

Since 1996, Ethiopia has been implementing polio eradication initiative activities using standard World Health Organization(WHO) recommended strategies [4], with significant success recorded, resulting in interruption of transmission of indigenous wild poliovirus (WPV) in December 2001, just five years after launching the “Kick polio Out of Africa” campaign [5]. However, the country has experienced numerous separate WPV importations from neighboring countries, including the 2013 polio outbreak, which was genetically linked to the virus circulating in neighboring countries, Somalia and Kenya, and related to the virus circulating in West Africa [6–8]. Ethiopia has been conducting case-based surveillance for Acute Flaccid Paralysis(AFP) countrywide since 1997 [9], and it is mandatory for all surveillance sites in the country to notify any suspected cases of AFP that fulfill the national case definitions including weekly zero case reporting. On top of this the case-based surveillance performance system is frequently reviewed and regular feedback provided using the main global surveillance performance indicators to all partners at national and sub-national level, including to the WHO African Region and Ineter Country Support Team(IST), and inaddition an in-depth external review is conducted every 2 to 3 years by external assessors.

We report the results of a retrospective analysis conducted to describe the characteristics of AFP surveillance performance, progress and challenges, and chachetrstics of reported AFP cases, between 2005 and 2015 to evaluate the case based surveillance performance using the WHO and nationally recommended surveillance standards, in order to review challenges and draw lessons learned.

## Methods

**Study area:** Ethiopia is one of the most populous countries in Africa with an estimated total population of over 91 million as per 2007 population census projection. Administratively the country is subdivided into nine Regional States and two City Administrations, which are further sub-divided into zones, woredas (which are equivalent to districts) and Kebeles.

**Study design:** we conducted a descriptive study design to make use of secondary data reported to the WHO Ethiopia Country Office surveillance database.

**Study population:** all children below15 years old with sudden onset of weakness or floppiness in one or more limbs and any adult above 15 years old that a clinician suspects polio.

**Investigation of cases:** initial investigations of suspected cases were done by health workers, using a standard case investigation form to capture demographic, clinical and epidemiological information. Additionally, WHO surveillance officers validated at least 80% of the cases and conducted a detailed investigation for all late and inadequate cases. Furthermore, the National Polio Expert Committee (NPEC) composed of personnel with different expertise classified cases upon submission of full documentation.

**Laboratory methods:** when a case was suspected two stool samples were collected and transported to the WHO accredited national polio laboratory where the global and national guidelines for testing samples was followed.

**Data collection and analysis:** we used the case based data from 2005-2015, which was entered into an MS Access database on a daily basis for program monitoring purposes in the WHO country office. We converted the MS Access database into MS-Excel, and into Epi Info version 3.5.1 (Centers for Disease Control and Prevention, Atlanta, United States), where all descriptive analysis frequencies, tables, and graphs were generated.

**Ethical approval:** the WHO Country Office and Ethiopian Public Health Institute gave ethical approval for analyzing and publishing the data.

### Definitions of terms

**Suspected AFP:** any child below 15 years of age with weakness or floppiness of one or more limbs or any person of any age in whom a clinician suspects polio.

**Confirmed polio case:** a suspected case with WPV isolation from a stool sample.

**Non-polio AFP cases:** discarded cases or all AFP cases excluding WPV and compatible cases.

**Non-polio AFP rate** = Number of non-polio AFP cases < 15 years old X 100,000/ Total number of children < 15 years old

**Stool adequacy rate** = Total # AFP cases with 2 stool specimens within 14 days of onset of paralysis * 100/ Total AFP cases reported

## Results

The current study reports the results of surveillance for non-polio AFP cases that were investigated between January 2005 and December 2015. Between these periods a total of 11728 AFP cases were reported, of which 7,037(58.1%) were male, and the majority 7994(68.2%) were below 5 years old. The mean age was 5.1 years with standard deviation (SD) ±3.8 years (Table 1). Fever at onset of paralysis was record for 8302 and since 2007 on average 86.5% of the cases developed fever at onset of paralysis ranging from 84.3% to 89.6%. The mean number of days from onset of paralysis to notification of AFP cases was 7.4 days ranging from 6.0 days to 9.2 days, and more than 95% of the cases were investigated within less than 2 days from notification. The mean number of days from notification to second stool specimen collection was 2.4 days (range from 1.9 to 3 days), while the mean days from second stool collection to laboratory arrival was 1.6 (range from 1.4 to 1.9 days).

**Table 1:** Characteristics of Acute Flaccid Paralysis cases, 2005-2015, Ethiopia

Parameter	2005	2006	2007	2008	2009	2010	2011	2012	2013	2014	2015	average
AFP cases reported	950	815	910	1098	1001	1110	1080	1183	1189	1213	1179	11728
%Male(N=7037)	60	56.6	57.6	58.4	57.1	60.4	53	59	59.4	57.6	60	58.1
%Female(n=4691)	40	43.4	42.4	41.6	42.9	39.6	47	41	40.6	42.4	40	41.9
% 0-5 Years(n=7994)	73.3	75.8	70.6	74.1	69.7	66.9	64.5	63.7	66.8	61.9	62.5	68.2
% 6-9 Years(n=2280)	17.4	14	19.1	17.6	17.7	18.7	21.8	23.5	21.1	23.6	19.3	19.4
% 10-15+ years(n=1462)	9.3	10.2	10.3	8.1	12.6	14.4	13.6	13.7	12.2	14.5	18.2	12.5
% Fever at onset of paralysis (n=8302)	0	0	85.9	89.6	89	87	86.6	85.2	84.8	86.3	84.3	86.5
% asymmetric paralysis (n=5951)	62.5	65.0	63.2	47.7	44.1	50.3	41.9	40.7	42.5	39.6	60.6	50.8
% paralysis progressed within 3 days(n=9001)	78.7	75.7	77.4	81.1	80.7	77	80.4	76.4	72.6	71.7	72.5	76.7
% True AFP cases after investigation(n-11643)	100.0	100.0	100.0	99.1	100.0	99.9	99.1	99.0	98.7	98.7	97.5	99.3

**Figure 1:**
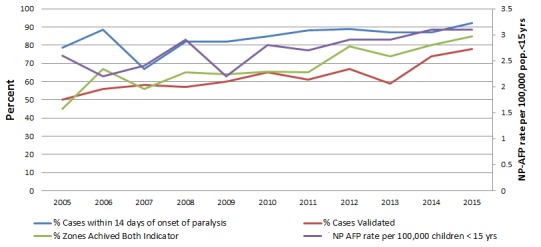
Trends of main surveillance performance indicators 2005-2015

The proportions of stool specimens arriving in good condition in the laboratory throughout the study period were above the minimum expected target of 90% except in 2010, 2013 and 2014, when there was a decline to 88.3%, 81.6%, and 80% respectively. On the contrary, the non-polio enterovirus isolation rate has been declining since 2010, and the minimum standard (10%) was not maintained except in 2005, 2007 and 2009 (Table 2). The national non-polio AFP and the stool adequacy rates substantially improved and were maintained above certification level in all the years that were reviewed with the exception in 2005 and 2007, where the stool adequacy rate was below standard (Figure 1).

**Table 2:** Case investigation, sample transportation and classification of cases by year 2005-2015, Ethiopia

Parameters	2005	2006	2007	2008	2009	2010	2011	2012	2013	2014	2015	average
Onset to notification in days	9.2	7	7.2	7.7	8.1	7.4	7	6.9	7.9	7.3	6	7.4
Mean Days to notification to second stool	3	2.4	2.6	2.4	2	1.9	2.2	2.2	2.3	2.4	2.5	2.4
% Investigated < 2 days of notification	89.9	96.7	95.6	95.2	98.1	98.5	97.7	96.8	95.4	96.2	94	98.5
Second stool to lab arrival in days	1.4	1.4	1.5	1.4	1.6	1.7	1.9	1.6	1.6	1.6	1.6	1.6
%Specimen arriving at lab within 3 days	98.8	98.8	98.5	99.2	99	99.1	98.3	98.6	98.3	96.9	98	98.5
% of Specimen arriving in good condition	99.4	98.9	99.6	91.4	99.7	88.3	91.4	91.5	81.6	79	80	91
% of NPENT cases isolated	12	7	11.6	8.3	10.6	6.5	6.9	4.5	9.2	7	3.2	7.9
%Suspected Polio Virus Isolation Rate	4	5	5.4	3.3	3.8	7.8	2.2	1.2	7.3	8	4.5	4.4
Confirmed WPV cases	22	17	0	3	0	0	0	0	9	1	0	52*
Compatible	25	26	15	11	30	38	17	15	37	17	1	232*
Discarded	903	772	895	1079	969	1055	1039	1139	1119	1179	1164	1313*
None AFP cases	0	0	5	0	0	11	11	24	19	14	14	98*

The national non-polio AFP rate ranged from 2.2 -3.1/100,000, children < 15 years with an increase from 2.6 in 2005 to 3.1 by the end of 2015, while the stool adequacy rate increased from 78.5% in 2005 to 92% in 2015, and ranged from 67% - 92% (Figure 1). Although there had been a remarkable improvement, it had been below operational standard in 2005 and 2007.

All the 11 Regions reported AFP cases; the majorities were from Oromia (36.8%), SNPPR (21.9%) and Amhara (21.6%) Regions. However, in terms of overall performance, only South Nations and Nationalities People's Region(SNNPR) achieved and maintained the two main AFP surveillance performance indicators above certification level, while Addis Ababa, Afar, and Tigray failed to achieve non-polio AFP rate in 2013, and Amhara in 2006, while Benshangule- Gomuz, Oromia, Dire Dawa and Gambella regions Have achieved the minimum expected None Polio AFP (NP-AFP) rate all the years' reviewed. Nevertheless, the stool adequacy rate in Oromia was lower than expected in 2005, and Dire Dawa in 2005 and in 2010. On the other hand, Gambella, Harari, Benshangul-Gumuz, and Somali Regions did not achieve and maintain the two main surveillance indicators particularly the stool adequacy rate.

**Figure 2:**
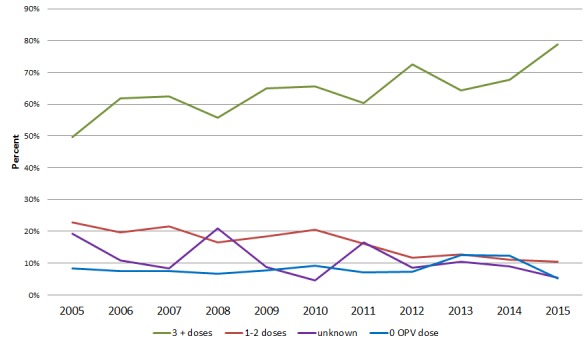
Immunization status of reported AFP cases 2005-2015, Ethiopia

**Figure 3:**
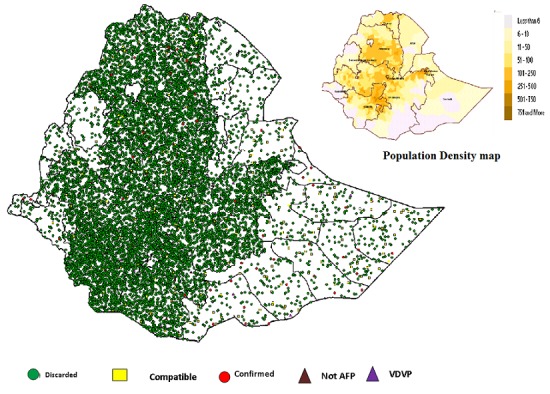
Final classification of AFP case in relation of population density 2005-2015, Ethiopia

The surveillance performance indicators in these regions fluctuated from year to year and did not show a discernible trend except Somali and Harari Regions, which was persistently low (Table 3). In Somali Region, both surveillance performance indicators were below expected standard for almost all the years analyzed except in 2005 and 2014(Table 3). The mean proportion of AFP cases with zero doses of Oral Polio Vaccine (OPV) was 5%, while the proportion of cases who had received three or more doses of OPV showed a steady increase over the study period from 50% to 79 % (Figure 2). A total of 53 WPV type 1 cases were detected between 2005 and 2006, and 10 cases between 2013 and 2014, all cases were attributed to importations. Of the total AFP, cases notified 11313 (96.5%) of the cases were discarded, while 233 (2.0%) were classified as compatible (Table 2 and Figure 3).

**Table 3:** Main surveillance indicators by Region, 2005-2015

Regions	Indicators	2005	2006	2007	2008	2009	2010	2011	2012	2013	2014	2015
**Addis Ababa**	NP-AFP rate	2.5	2.1	3.1	2.2	2.1	2.4	2.1	2.1	1.6	3	2.4
	St. Adequacy	81	97	96	94	94	94	97	94	82	90	88
**Afar**	NP-AFP rate	2.4	2.3	3.6	4.3	4	4.7	2.6	2.6	1.8	3.8	4
	St. Adequacy	94	94	88	73	93	97	90	88	100	83	92
**Tigray**	NP-AFP rate	5	3.4	2.6	2.6	2.7	2.8	2.6	2.6	1.9	2.6	2
	St. Adequacy	76	96	93	96	89	92	89	98	88	90	87
**Amhara**	NP-AFP rate	2.2	1.6	2	2.6	2.5	3	2.4	2.4	2.9	2.7	3.5
	St. Adequacy	79	92	83	85	79	87	75	87	86	85	92
**Benshangule Gumuz**	NP-AFP rate	6	3.3	3.6	4	3	4	4	3	4.4	4.4	3.8
	St. Adequacy	92	88	71	88	78	67	83	70	71	85	76
**Oromia**	NP-AFP rate	2.4	2	2.3	3.3	3	2.6	2.5	2.8	2.7	3	2.9
	St. Adequacy	73	90	87	87	86	85	88	90	88	88	91
**SNNPR**	NP-AFP rate	2.6	2.5	3	3	2.6	3.1	3	3	2.7	2.9	2.6
	St. Adequacy	92	92	93	93	93	94	96	96	92	93	97
**Dire Dawa**	NP-AFP rate	3.5	2.5	2	3.5	0.5	2	2.5	2.5	3	2	4
	St. Adequacy	71	100	100	100	100	75	100	89	100	100	100
**Gambella**	NP-AFP rate	11	2	2	10	7	2.5	3.5	3.5	2.8	2	2
	St. Adequacy	45	100	100	54	86	60	88	70	88	69	100
**Hareri**	NP-AFP rate	9	12	5	4	1	3	5	5	2	5	3
	St. Adequacy	100	92	100	75	50	75	60	100	50	67	100
**Somali**	NP-AFP rate	2	2.4	1.8	1.5	1	1.3	1.2	1.2	3.4	5	4.3
	St. Adequacy	84	70	76	79	72	61	73	60	62	74	92

The table 3 shows that all regions except Addis Ababa, Tigray, Dire Dawa, Hareri and Somali have achived the NP-AFP rate since 2005, however only two regions have achived stool adeqcy rat within the period of analysis.

## Discussion

Our analysis provides evidence that AFP surveillance performance indicators progressively improved at the national level over the years. The highest number of AFP cases was reported in Oromia, Amhara, and SNNPR Regions, which may be due to the size of the population of these regions as they are contributing to more than 80% of the country's population [10]. In addition, these regions have a better infrastructure, a relatively well organized and well-staffed health delivery system, which may be a reason for increased surveillance sensitivity. Sensitive surveillance is critical not to miss undetected circulation, and investigate and collect specimen as quickly as possible. There were fluctuations below standard in stool adequacy rate in 2005 and 2007at national level and in the achievement of key performance indicators at Regional level, notably in pastoralist Regions (i.e. Somali, Gambella, and Benshangul-Gumuz) and Harari region. This may be attributed to the nomadic lifestyle, hard to reach, insecure areas and understaffed health delivery system.

The health sector development program (HSDP IV) also reflected the lack of appropriate health service delivery package in pastoralist regions [11]. We also found that the non-polio enterovirus isolation rate was persistently low since 2010 and this may be due to low cell and reference virus cells sensitivity in the laboratory and issues with the reverse cold chain system from stool collection to arrival at the laboratory. Studies conducted in other countries indicate a higher rate of non-polio enterovirus isolation rate than what we found [12]. However, a study in Kurdistan Province, Western Iran' indicated a lower rate which is consistent with our findings [13] .

Vaccination coverage with three or more doses of OPV among AFP cases was 79%. This may indicate, besides other explanations, that the actual vaccine coverage is lower than the expected population immunity to prevent outbreaks, which may be even worse in pastoralist regions. Evidence from the national demographic health survey conducted in 2011 indicates polio three coverage of 44.3%, even less than what we found [14], while other similar studies conducted elsewhere indicate a higher rate [15].

In addition, we found that the sex distribution of AFP cases reported over the years have no significant difference among boys (58.1%) and girls (41.9%) (p>0.05), a study done in Nigeria showed a higher rate for females than boys, while. AFP cases below 5 years old to be 68.2%, which is lower than some studies done elsewhere, which found a higher rate [16, 17], but still our result is higher and consistent with other studies [18, 19]

Our study had a number of limitations. First due to data incompleteness, we used a dataset from 2005 onwards. Second, some variables were missing, so to try and mitigate we excluded cases with missing variables and used only those cases with required information available for analysis. Despite the limitations, our study highlights important strengths and aspects for improvement of the AFP surveillance system in the country in order to keep on the tracks towards certification of polio eradication. Secondly, missing data for some variables indicates a need for data improvement.

## Conclusion

In conclusion, we found that the AFP surveillance system was effective over the 10 years period between 2005 and 2015 in Ethiopia; meeting the surveillance performance indicators at national and subnational level. However, the sub-national performance in some regions remains a concern, particularly in pastoralist regions. We also discovered that the non-polio enterovirus isolation rates have been declining since 2010, and the stool condition didn´t meet the expected minimum (90%) in 2010, 2013, 2014 and 2015. Nevertheless, major strengths of the surveillance system include timely detection and transportation of specimen from corners of the country even in remote and hard to reach areas. We recommend that the two key AFP surveillance indicators be strengthened further to better enhance the overall performance, especially in pastoralist Regions (Somali, Gambella, and Benshangul-Gumuz). We also recommend that in view of the prevailing risk of wild poliovirus importation, community-based surveillance is intensified with a focus on pastoralist regions and border areas in order to increase the sensitivity of the surveillance system and to provide a high degree of confidence for timely detection of importation and any emerging event. Finally, we recommend an operational study be conducted aimed at improving the low non-polio enterovirus isolation rate, including a capacity review of laboratory sensitivity and the reverse cold chain system.

### What is known about this topic

- Ethiopia eradicated indigenous polio in 2001;
- Acute flaccid paralysis is one of the polio eradication strategies that country implementing;
- Surveillance performance indicators being monitor to achieve polio eradication.

### What this study adds

- The study provides valuable information on surveillance performance over years comparing with regional and global standards;
- The study also identifies challenges and progress, and recommends ways of improving the surveillance performances for certification;
- The study also indicates the focus needs to high risk and hard to reach areas to improve.

## Competing interests

All authors declare that there are no competing interests. The views expressed in the perspective articles are those of the authors alone and do not necessarily represent the views, decisions or policies of the institutions with which they are affiliated and the position of World Health Organization.
